# SHP2 is induced by the HBx-NF-κB pathway and contributes to fibrosis during human early hepatocellular carcinoma development

**DOI:** 10.18632/oncotarget.15930

**Published:** 2017-03-06

**Authors:** Hyo Jeong Kang, Dal-Hee Chung, Chang Ohk Sung, Su Hyun Yoo, Eunsil Yu, Nayoung Kim, Sy-Hye Lee, Ji-Young Song, Chong Jai Kim, Jene Choi

**Affiliations:** ^1^ Department of Pathology, Asan Medical Center, University of Ulsan College of Medicine, Seoul, Korea; ^2^ Asan Institute for Life Science, Asan Medical Center, Seoul, Korea

**Keywords:** SHP2, NF-κB, hepatitis B virus, cirrhosis, hepatocellular carcinoma

## Abstract

The non-receptor tyrosine phosphatase SHP2 has scaffolding functions in signal transduction cascades downstream of growth receptors. A recent study suggested that SHP2 acts as a tumor suppressor during hepatocellular carcinoma (HCC) development. Herein we examined whether SHP2 links the HBx–NF-κB pathway to EGFR signaling during HCC development. The overexpression of HBx or NF-κB led to increased SHP2 expression via NF-κB binding to the *Shp2* promoter. EGF treatment induced ERK activation as well as the rapid assembly of SHP2, EGFR, and Gab1. Upon LPS stimulation, NF-κB–SHP2–ERK activation and phosphorylated STAT3 levels exhibited a negative correlation *in vitro*. By contrast, in patients with HBV-associated HCC, NF-κB–SHP2–ERK and IL-6–JAK–STAT3 pathway activity levels were concomitantly higher in adjacent non-neoplastic tissues than in HCC tissues. The immunohistochemical analysis of 162 tissues of patients with HCC revealed that SHP2 levels were significantly higher in non-neoplastic background tissues than in corresponding HCC tissues and considerably increased in background liver tissues with advanced fibrosis (*P* < 0.001). SHP2 expression increased gradually from normal liver to chronic hepatitis, cirrhosis, and background liver with a dysplastic nodule, but was decreased or lost in dysplastic nodules and HCC. This is the first report to describe the existence of the HBx–NF-κB–SHP2 pathway, linking HBV infection to the EGFR–RAS–RAF–MAPK pathway in the liver. SHP2 depletion from the negative crosstalk between NF-κB and STAT3 accelerates HCC development.

## INTRODUCTION

Worldwide, hepatocellular carcinoma (HCC) is among the most common types of cancer and the second leading cause of cancer-related deaths. Chronic infection with hepatitis B virus (HBV) or hepatitis C virus (HCV) accounts for up to 80% of HCC cases [1].

HBV infection causes the immune-mediated destruction of infected hepatocytes and subsequent liver regeneration, which are associated with liver fibrosis, cirrhosis, and HCC [2, 3]. The precise mechanism linking HBV infection to HCC is not entirely understood, especially as HBV is considered to be only weakly carcinogenic [4, 5]. Continual immune-mediated HBV clearance and increases in inflammatory cytokine levels have been suggested to activate cell proliferation and survival signals [6]. HBV X protein (HBx), a 154-amino-acid protein with a molecular mass of 17 kDa, is the major contributor to the long-term disease process of chronic active hepatitis. Rather than directly binding to DNA, HBx protein indirectly activates transcription factors such as NF-κB, activator protein-1 (AP-1), ATF/CREB, proto-oncogenes (c-myc and c-jun), and HIF-1 [7–12]. HBx also promotes cell proliferation by activating the RAS–RAF–MAPK pathway via associations with RAS, Shc, Grb2, and SOS, thereby stimulating RAS–GDP loading [13, 14]. Although many reports have described HBx activities, the molecular mechanism linking HBx activity to HCC remains poorly understood.

Nuclear factor-kappa B (NF-κB), a dimeric transcription factor, comprises a family of proteins, including NF-κB1 (p105 and p50), NF-κB2 (p100 and p52), RelA (p65), c-Rel, and RelB [15, 16]. NF-κB normally localizes to the cytoplasm and binds to members of the inhibitory IκB family (IκBα, IκBβ, p105, and p100), blocking the nuclear translocation of NF-κB [6]. To activate NF-κB, HBx forms complexes with IκBα in the cytoplasm or reduces the levels of cytoplasmic p105 proteins, leading to the nuclear accumulation of NF-κB and activation of the NF-κB pathway [17, 18]. NF-κB acts dual pro-inflammatory and anti-apoptotic roles in combating hepatocellular injury [6].

SHP2, encoded by *Ptpn11*, is a Src homology 2 domain-containing non-receptor tyrosine phosphatase and a proto-oncogene. Autosomal-dominant mutations in *Ptpn11*, which have been detected in approximately half of the patients with Noonan syndrome, predispose affected individuals to juvenile myelomonocytic leukemia and several other neoplasms that exhibit increased SHP2 phosphatase activity [19–21]. SHP2 overexpression has been reported in leukemia and breast cancers [22, 23]. Earlier studies have shown that SHP2 promotes EGFR–RAS–RAF–MEK–ERK pathway activity by dephosphorylating the RAS-GTPase-activating protein (RAS–GAP) binding site on the docking protein Grb2-associated binder 1 (Gab1), leading to prolonged RAS activation [24, 25].

A recent study reported that hepatocyte-specific SHP2 deletion promotes hepatic inflammation and necrosis, leading to regenerative hyperplasia, fibrosis, and HCC development [26]. SHP2 knockout was found to greatly increase signaling strength in the inflammatory IL-6–signal transducer and activator of transcription 3 (STAT3) pathway. In contrast, STAT3 ablation completely alleviates the effect of SHP2 depletion on HCC development. Therefore, SHP2 is a tumor suppressor that may counteract STAT3 activity in the liver. The contrasting roles of SHP2 in leukemogenesis and liver cancer suggest that SHP2 activity must be finely maintained [27]. Here we report for the first time the existence of an HBx–NF-κB–SHP2 pathway in HBV-infected livers. We found that HBx increased SHP2 expression through NF-κB, leading to prolonged EGFR signaling. Moreover, SHP2, NF-κB, and phospho-STAT3 levels were simultaneously higher in non-neoplastic background tissues than in corresponding HCC tissues. In an immunohistochemistry (IHC) analysis of 162 HCC tissues, SHP2 expression was decreased or absent in tumor tissues from a significant fraction of patients with HCC. By contrast, higher SHP2 expression levels were observed in the background liver tissues of patients with advanced fibrosis. In addition, SHP2 expression gradually increased from normal liver to HBV-associated cirrhotic liver, but decreased in dysplastic nodules. We also present evidence that SHP2 links HBx–NF-κB with EGFR signaling-mediated hepatocyte proliferation and that the HBx–NF-κB–SHP2 and IL-6–JAK–STAT3 pathways were active in parallel at an early stage of HCC development; accordingly, losses of activity in these pathways could accelerate HCC development.

## RESULTS

### HBx increases SHP2 mRNA and protein levels and stimulates growth signaling

**Figure 1:**
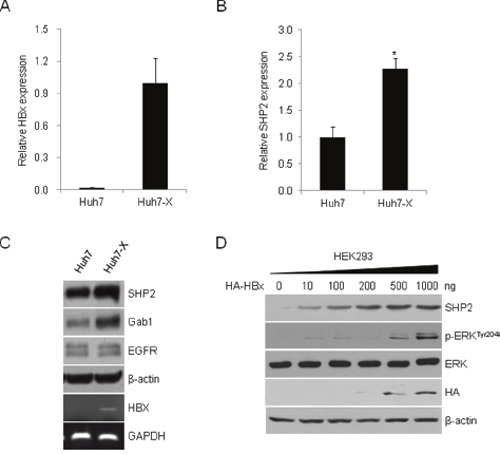
HBx induces SHP2 expression and activates ERK signaling HBx **(A)** and SHP2 **(B)** transcript levels in Huh7 and Huh7-X (HBx-overexpressing) cells were analyzed by qRT-PCR. Data indicate the mRNA levels of HBx and SHP2 relative to that of the control gene, GAPDH. Significance *vs*. control, *, *P* < 0.05 (n = 3). **(C)** SHP2, Gab1, and EGFR protein levels were analyzed in Huh7 and Huh7-X cells by Western blotting with the indicated antibodies. *HBx* expression in Huh7-X cells was determined by RT-PCR. GAPDH was used as a control. **(D)** HEK293 cells were transfected with increasing amounts of HA-tagged HBx expression vectors. After 48 h, Western blotting was performed using the indicated antibodies.

Because SHP2 can act as both a proto-oncogene and tumor suppressor [27], we first questioned whether SHP2 is activated by oncogenic HBx in HCC cells. HBx and SHP2 transcript levels were measured in Huh7 and stably HBx expressing Huh7-X cells. Both the HBx and SHP2 mRNA levels were markedly higher in Huh7-X cells than in Huh7 cells (Figure 1A, 1B). SHP2 protein levels were also higher in Huh7-X cells than in Huh7 cells (Figure 1C). The expression of Gab1, an adaptor protein that recruits SHP2 to the EGFR signaling cascade, was slightly higher in cells with increased SHP2 levels. To examine the relationship between HBx and SHP2 overexpression, we transfected non-hepatic human embryonic kidney (HEK) 293 cells with increasing amounts of the HA–HBx expression vector. As shown in Figure 1D, the levels of SHP2 and its downstream effector, phospho-ERK, were dose-dependently increased with the amount of transfected HA–HBx. These data suggest that HBx activates SHP2 expression.

### HBx induces SHP2 expression via NF-κB–p65

**Figure 2:**
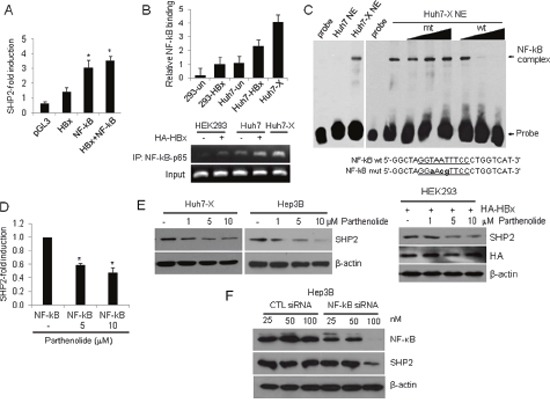
HBx activates the *Shp2* promoter via an NF-κB–p65 binding site **(A)** SHP2 promoter activities in HEK293 cells after transfection with Shp2–Luc and plasmids expressing HBx, NF-κB, or a combination of both for 48 h. Luciferase activity levels were normalized to *Renilla* luciferase units. Significance *vs*. control, *, *P* < 0.05 (n = 6). **(B)** ChIP analysis of NF-κB–p65 binding to the *Shp2* promoter after HA–HBx overexpression for 24 h. **(C)** EMSA was performed with Huh7 and Huh7-X nuclear extracts using biotin-labeled probes containing the NF-κB binding site on the *Shp2* promoter. Competition assays were performed using 1-fold, 10-fold, and 100-fold molar excesses of unlabeled wild-type (wt) and mutant (mut) oligonucleotides. **(D)** *Shp2* promoter activities in NF-κB-overexpressing HEK293 cells after parthenolide treatment for 18 h. **(E)** Western blot analyses of SHP2 expression in Huh7-X, Hep3B and HEK293 cells after treatment with parthenolide. **(F)** Hep3B cells were transfected with a control siRNA or a NF-κB siRNA. After 48 h, the cells were analyzed by Western blot with anti-NF-κB and anti-SHP2 antibodies.

Because HBx stimulates cell growth-related transcription by indirectly activating transcription factors [7, 9], we used the Transcription Element Search System to analyze a 3,000-bp region around the *Shp2* promoter and identify *cis*-regulatory elements that might explain the effect of HBx on SHP transcription [28]. We identified a putative NF-κB binding site between nucleotides −510 and −500 (GGTAATTTCC; numbering starting at the translation start site) and cloned a fragment from the 5′-flanking region of the *Shp2* promoter (spanning nucleotides −1,727 to −151) to the upstream region of the luciferase reporter gene to yield a Shp2–Luc construct. As the HBx protein induces NF-κB activation in liver cells, we first examined the effect of HBx or NF-κB on *Shp2* expression. As shown in Figure 2A, HBx or NF-κB overexpression significantly increased *Shp2* promoter activity up to 2- or 4- fold, respectively. Expression from the *Shp2* promoter was further increased by approximately 5-fold in HBx and NF-κB co-transfected cells. We performed a chromatin immunoprecipitation (ChIP) assay using antibodies against p65 (RelA), an NF-κB protein. The results showed a significant increase in endogenous NF-κB–p65 binding to the *Shp2* promoter after HBx overexpression in both HEK293 and Huh7 cells (Figure 2B). To investigate whether NF-κB directly binds to the *Shp2* promoter, we performed electrophoretic mobility shift assay (EMSA) using nuclear extracts prepared from Huh7 and Huh7-X cells. We found a shifted band with Huh7-X, but not with Huh7. The shifted band was eliminated by the addition of 10-fold or 100-fold excess of the same unlabeled NF-κB binding DNA of the *Shp2* promoter. In contrast, mutated NF-κB probe were unable to out-compete the complexes (Figure 2C).

To further confirm the effect of HBx–NF-κB pathway activity on *Shp2* expression, Shp2 promoter activity was measured after treatment with increasing amounts of the NF-κB inhibitor parthenolide. The results revealed that both *Shp2* promoter activity and SHP2 protein levels were dose-dependently reduced in Huh7-X, Hep3B and HEK293 (Figure 2D, 2E). Next, we assessed the effects of NF-κB knockdown on SHP2 expression in Hep3B cells. Transfection of a NF-κB siRNA resulted in a marked decrease in SHP2 protein levels compared with transfection of a control siRNA (Figure 2F). These data confirm that HBx induces *Shp2* expression at the transcription level via the HBx–NF-κB–SHP2 pathway in HBV-infected liver cells.

### SHP2 acts as a link between HBx and RAS signaling and a negative regulator of STAT3 signaling in HCC cells

**Figure 3:**
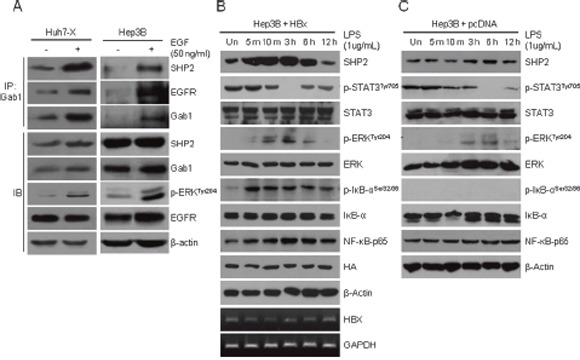
EGFR–SHP2–Gab1 complex-mediated ERK signaling and STAT3 signaling are inversely active in hepatocellular carcinoma cells **(A)** A ternary complex comprising SHP2, EGFR, and Gab1 was immunoprecipitated (IP) using anti-Gab1 antibodies after EGF treatment for 10 min. Endogenous levels of SHP2, Gab1, EGFR, and phosphorylated ERK proteins were determined by immunoblotting (IB). **(B)** HBx-overexpressing Hep3B cells were stimulated with LPS (1 μg/mL). The relative amounts of total SHP2, p-STAT3, STAT3, p-ERK, ERK, p-IKB, NF-κB-p65 and HA were determined by Western blotting at the indicated times. β-Actin was used as the loading control. The expression of the *HBx* gene was detected by RT-PCR. GAPDH served as a control. **(C)** Hep3B cells were stimulated with LPS (1 μg/mL) and analyzed as described in panel **(B)**.

Numerous studies have reported that HBx increases the levels of GTP-bound RAS [13, 14, 29]. However, the mechanism by which HBx activation mediates the RAS pathway is not fully understood. SHP2 competes with RAS–GAP, a terminal protein of the EGFR pathway, for binding to Gab1. We hypothesized that in HBV-positive HCC cells, SHP2 overexpression induced by HBx–NF-κB pathway activation extends RAS activation. To test whether EGF stimulation alters SHP2 binding to the EGFR–Gab1 complex, we treated Huh7-X and Hep3B cells with EGF. EGF-treated Huh7-X and Hep3B cells exhibited the rapid assembly of a ternary complex comprising SHP2, EGFR, and Gab1, as well as ERK activation (Figure 3A). The hepatic inflammatory response activates the IL-6 and STAT3 pathway, accelerating HCC development [30]. Based on this notion, we next treated HBx-overexpressing Hep3B cells with LPS to determine the cell-intrinsic inflammatory responses. As shown in Figure 3B, NF-κB, SHP2, and phospho-ERK levels increased within 5 min of LPS treatment and peaked at 3 h. In contrast, phosphorylated STAT levels were lowest at 3 h, and increased thereafter. We further analyzed the inflammatory response to LPS in Hpe3B cells without HBx overexpression. The induction pattern of the SHP2-ERK pathway and STAT3 signaling was similar to that of HBx overexpressing Hebp3B (Figure 3C); however, SHP2 induction and complete STAT inactivation were noticeably delayed to 6 h in LPS-stimulated Hep3B cells. These results suggest that SHP2 is the missing link between the HBx–NF-κB pathway and RAS signaling. Moreover, an inverse relationship between the HBx–NF-κB–SHP2 pathway and STAT3 signaling exists in HCC cells *in vitro*.

### High expression of SHP2 and NF-κB–p65 proteins in HBV-infected liver tissues

**Figure 4:**
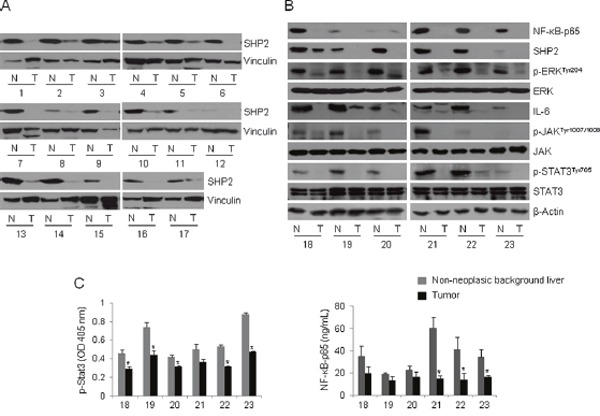
The NF-κB–SHP2–ERK and IL-6–JAK–SHP2 pathways are concomitantly increased in HBV-associated liver tissues **(A)** Whole tissue lysates were prepared from 17 pairs of HBV-infected tumors (T) and matched non-neoplastic tissues (N), and analyzed by Western blot with anti-SHP2 antibodies. Vinculin served as a loading control. **(A)** NF-κB-p65, SHP2, p-ERK, ERK, IL-6, p-JAK, JAK, p-STAT3 and STAT3 levels were assessed by Western blotting in T/N tissues prepared as described in A. β-Actin was used as a loading control. **(B)** The whole tissue lysates described in **(A)** were assayed to determine the levels of p-STAT3 and NF-κB-p65. Columns represent the means of three independent experiments; bars indicate standard deviations (* *P* < 0.05). The numbers denote tissue numbers.

Formation of the Gab1/SHP2 complex is crucial for liver regeneration. Liver-specific SHP2 or Gab1 knockout mice display defective liver regeneration after partial hepatectomy [31]. Jiang *et al*. reported that SHP2 acts as a tumor suppressor protein and that decreased SHP2 expression is a poor prognostic marker in HCC [32]. To further confirm the existence of the HBx–NF-κB–SHP2 pathway, we obtained 23 pairs of tumor tissues and surrounding non-neoplastic tissues from patients with HBV-associated HCC and analyzed SHP2 expression via Western blot analysis. SHP2 expression was significantly lower in tumor tissues compared with surrounding non-neoplastic tissues in the 17 pairs examined (Figure 4A). We further analyzed 6 pairs of tissues in terms of the activity of the NF-κB–SHP2 and STAT3 signaling pathways. Notably, HCC-surrounding non-neoplastic tissues expressed significantly higher levels of NF-κB–p65, SHP2, and phospho-ERK than those of matched HCC tissues (Figure 4B). Bard-Chapeau *et al*. reported that increased inflammatory signaling through STAT3 could exacerbate chemical-induced HCC development [26]. In addition, HBV infection greatly exacerbates the NF-κB-mediated host immune response, and active NF-κB subsequently triggers massive inflammatory responses via IL-6–JAK–STAT3 signaling [33, 34]. Based on these observations, we next examined STAT3 activation in patient tissue samples. In contrast to *in vitro* data suggesting an inverse relationship between HBx–NF-κB–SHP2 activity and STAT3 signaling, HCC-surrounding non-neoplastic tissues exhibited increased IL-6–JAK–STAT3 signaling compared with matched tumor tissues. Decreased levels of both phospho-STAT3 and NF-κB in tumor tissues were also confirmed by enzyme-linked immunosorbent assay (ELISA; Figure 4C). These data suggest that the anti-parallel NF-κB–SHP2 and STAT signaling pathways are competitively active in precancerous inflammatory liver tissues, but their activities are lost simultaneously upon transformation of liver cells during HCC development.

### High SHP2 expression in non-neoplastic livers with advanced fibrosis

**Figure 5:**
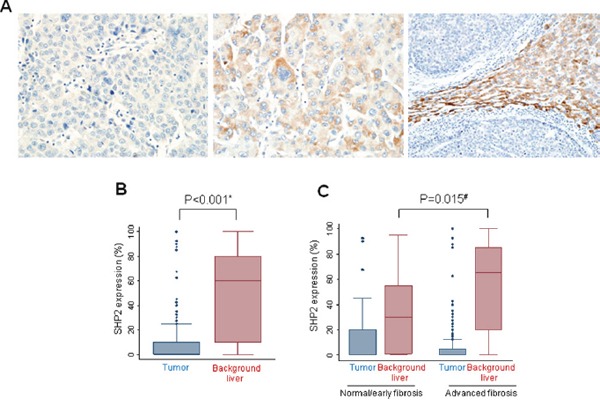
Immunohistochemical detection of SHP2 expression in human HCCs **(A)** SHP2-negative tumor cells (left), SHP2-positive tumor cells (middle), and SHP2-negative tumor cells on a SHP2-positive liver background (right). **(B)** SHP2 expression is lower in tumor cells than in matched non-neoplastic liver cells. **(C)** SHP2 expression tends to be higher in surrounding tissues with advanced fibrosis. *, Wilcoxon matched-pairs signed ranks test and #, Wilcoxon rank sum test.

Because our earlier results indicating a loss of SHP2 in HCC tissues relative to HBV-positive paired background tissues were not obtained from a random cohort, we performed an additional immunohistochemical analysis of SHP2 expression in 162 tissues from patients with HBV-associated HCC (Supplementary Table 1). Patient demographics are described in Table 1. SHP2 was strongly and diffusely expressed in the cytoplasm of both tumor cells and surrounding non-neoplastic liver cells in patients with HCC. Representative levels of SHP2 immunoreactivity in tumor cells and background liver cells are shown in Figure 5A. Notably, the SHP2 expression levels were lower in tumors than in matched non-neoplastic surrounding tissues (*P* < 0.001) (Figure 5B). In addition, SHP2-positive tumors tended to associate with a lack of microvascular invasion (*P* = 0.016) (Table 2). No associations were observed between SHP2 expression and other clinicopathologic factors including patient age, sex, etiology, tumor size, serum alpha-fetoprotein level, and histological grade. In the survival analysis, SHP2 positivity was not associated with overall (*P* = 0.092) or recurrence-free (*P* = 0.744) survival.

**Table 1:** Clinicopathologic characteristics of 162 patients with resectable hepatocellular carcinoma

Characteristics		Patients
	Number	%
All patients	162	100
Age (years)		
Median, years (SD)	53 (8.8)	
Range	26–71	
Sex		
Male	120	74.1
Female	42	25.9
Etiology		
HBV	162	100
BCLC stage		
1	152	93.8
2	10	6.2
Fibrosis stage		
Early (I-II)	26	16.0
Advanced (III-IV)	136	84.0
Tumor size		
<5 cm	121	74.7
≥5 cm	41	25.3
AFP level (ng/ml)		
Normal (<10)	57	35.2
Elevated (≥10)	105	64.8
Edmondson grade		
I-II	108	66.7
III-IV	54	33.3
Microvascular invasion		
No	112	69.1
Yes	50	30.9

SD, standard deviation; BCLC, Barcelona clinic liver cancer; AFP, alpha-fetoprotein

**Table 2:** Relationship between SHP2 expression and clinicopathologic parameters in 162 patients with surgically resectable HBV-associated hepatocellular carcinoma

Parameter	Total no.	SHP2 (−)	SHP2 (+)	*P*-value*
Age, years (median)	162	53	53	0.758
Sex (%)				
Male	120	85 (70.8)	35 (29.2)	0.201
Female	42	34 (81.0)	8 (19.0)	
Fibrosis stage (%)				
Early (I-II)	26	15 (57.7)	11 (42.3)	0.047
Advanced (III-IV)	136	104 (76.5)	32 (23.5)	
Tumor size (%)				
<5 cm	121	89 (73.5)	32 (26.5)	0.962
≥5 cm	41	30 (73.2)	11 (26.8)	
AFP level (ng/ml) (%)				
Normal (<10)	57	40 (70.2)	17 (29.8)	0.486
Elevated (≥10)	105	79 (75.2)	26 (24.8)	
Edmondson grade (%)				
I-II	108	75 (69.4)	33 (30.6)	0.102
III-IV	54	44 (81.5)	10 (18.5)	
Microvascular invasion (%)				
No	112	76 (67.9)	36 (32.1)	0.0168
Yes	50	43 (86.0)	7 (14.0)	

* *P*-value was calculated using the two-tailed Student's *t*-test.

HBV, hepatitis B virus; AFP, alpha-fetoprotein

When patients were divided into early fibrosis and advanced fibrosis groups, a tendency toward lower SHP2 expression was observed in tumor cells in the advanced fibrosis group than in tumor cells in the early fibrosis group (*P* = 0.045). Inversely, SHP2 expression in background liver cells was much higher in the advanced fibrosis group than in the early fibrosis group (*P* = 0.015, Figure 5C). These results strongly suggest that SHP2 expression is significantly associated with inflammatory responses leading to fibrosis during hepatocarcinogenesis.

### A gradual increase in SHP2 expression in accordance with increases in inflammation and fibrosis

**Figure 6:**
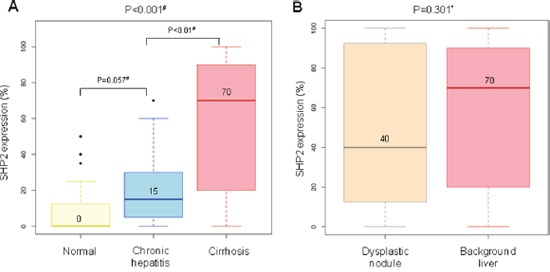
Representative levels of SHP2 immunoreactivity in livers without hepatocellular carcinoma **(A)** SHP2 expression tends to increase gradually from normal livers to cirrhotic livers. **(B)** SHP2 expression is lower in dysplastic nodules than in matched non-neoplastic cirrhotic liver tissues. *, Wilcoxon matched-pairs signed ranks test and #, Wilcoxon rank sum test. The number above the histogram represents the mean value.

To evaluate SHP2 expression according to the degrees of inflammation and fibrosis, tissue microarrays of 48, 21, and 51 patients with normal liver, chronic hepatitis, and dysplastic nodules, respectively, were subjected to IHC staining for SHP2 (Supplementary Table 2). The characteristics of the 120 patients are listed in Table 3. Normal liver tissues biopsied from healthy donors were negative for hepatitis A, B, and C viruses and chronic liver disease. All patients with HBV-associated chronic hepatitis exhibited variable degrees of inflammation and fibrosis, but not cirrhosis; however, HBV-positive patients with dysplastic nodules had cirrhotic background liver tissues. SHP2 expression was higher in chronic hepatitis tissues than in normal liver tissues (*P* = 0.057), and the highest levels observed in cirrhotic tissues (*P* < 0.001) (Figure 6A). In a further evaluation of cirrhotic tissues, SHP2 expression levels were lower in dysplastic nodules than in matched non-neoplastic cirrhotic background liver tissues, with mean values of 40 and 70, respectively (*P* = 0.301) (Figure 6B). Accordingly, the expression of phosphorylated STAT3 (active STAT3) was decreased in dysplastic nodules compared with chronic hepatitis tissues and completely lost in hepatocellular carcinoma tissue (Table 4), which is similar to the pattern of SHP2 expression. These data indicate a gradual increase in SHP2 expression during chronic liver inflammation and fibrosis, followed by a decrease during the early developmental stage of HCC.

**Table 3:** Clinicopathologic characteristics of patients without hepatocellular carcinoma

Characteristics	Normal (n = 48)	Chronic hepatitis (n = 21)	Dysplastic nodule (n = 51)
Mean age (range), years	26 (15–54)	47 (19–67)	54 (41–71)
Sex (%)			
Male	39 (81.3)	15 (71.4)	40 (78.4)
Female	9 (18.7)	6 (28.6)	11 (21.6)
Hepatitis B virus (%)			
Positive	0 (0)	21 (100)	51 (100)
Negative	48 (100)	0 (0)	0 (0)
AFP level (%)			
Normal (<10)	-	-	23 (45.1)
Elevated (≥10)	-	-	28 (54.9)
Cirrhosis (%)			
Present	0 (0)	0 (0)	51 (100)
Absent	48 (100)	21 (100)	0 (0)

AFP, alpha-fetoprotein

**Table 4:** Expression of p-STAT in HBV-infected liver

p-STAT	ChronicHepatitis	Dysplasticnodule	Hepatocelluarcarcinoma	*P*-value*
Positive	9 (42.8%)	2 (3.9%)	0 (0.0%)	<0.001
Negative	12 (57.2%)	49 (96.1%)	36 (100.0%)	

* *P*-value was calculated using the Fisher exact test.

p-STAT, phoshorylated STAT

## DISCUSSION

NF-κB acts major roles in liver damage induced by chronic inflammation and subsequent hepatocyte proliferation to replace damaged tissue [35–38]. Here we report for the first time the existence of an HBx–NF-κB–SHP2 pathway in the liver. The levels of SHP2 and NF-κB expression, as well as STAT3 phosphorylation, were significantly higher in non-neoplastic surrounding tissues than in matched HBV-associated HCC tissues. SHP2 expression gradually increased from normal liver tissues to those with chronic hepatitis and cirrhosis, decreased in dysplastic nodules, and was lost in HCCs. Of note, the combination of increased HBx–NF-κB–SHP2 pathway activity and increased STAT3 signaling in non-neoplastic background liver tissues suggests that these two signals are necessary for preventing HCC progression.

Gab1/SHP2 complex formation promotes ERK signaling by sequestering RasGAP, a RAS signal-terminating protein and thereby blocking its binding to Gab1 [39]. The Gab1/SHP2 complex can be detected immediately after hepatectomy, and liver-specific *Gab1* or *Shp2* knockout mice display similar defects in liver regeneration [31]. In our study, we found that the *Shp2* (*Ptpn11*) promoter contains a putative NF-κB binding site (5′-GGTAATTTCC-3′) located 500-bp upstream of the translation initiation site. Transiently expressed HBx or NF-κB induced *Shp2* promoter activity, and NF-κB bound to the *Shp2* promoter in the ChIP assay. These results are the first to establish a link between the HBx–NF-κB and EGFR–Gab1/SHP2–RAS pathways. Depending on the magnitude of NF-κB activity, SHP2 could promote the compensatory proliferation of surviving hepatocytes via ERK pathway activation in damaged hepatic tissues.

**Figure 7:**
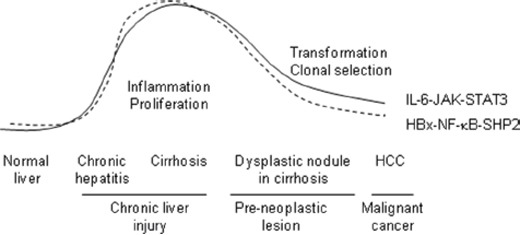
HCC-promoting HBx–NF-κB–SHP2 and IL-6–JAK–STAT3 pathways in the human liver The activity levels of both pathways concomitantly increase during chronic inflammation and then gradually decline during the malignant transformation of hepatocytes in dysplastic nodules. Constant crosstalk is observed between IL-6–JAK–STAT3-mediated inflammatory responses and compensatory hepatocyte proliferation via the NF-kB–SHP2–EGFR pathway after viral infection. SHP2 depletion skews this balance and leads to accelerated HCC development.

NF-κB activation after HBV infection leads to the secretion of pro-inflammatory cytokines, including IL-1β, TNFα, and IL-6, in hepatocytes. Among these, IL-6, an IL-6–JAK–STAT3 pathway component, has a specific and critical function in HCC development [34, 40]. In our experiments, we observed concomitant NF-κB and SHP2 overexpression in non-neoplastic tissues adjacent to HBV-associated tumors. Increased IL-6–JAK–STAT3 signaling was also detected in those tissues (Figure 4A, 4B). NF-κB activation is an early event in hepatocarcinogenesis [41]. The NF-κB-activating kinase IKKβ acts as an early suppressor of chemically induced liver tumorigenesis by inhibiting hepatocyte death and compensatory cell proliferation [42]. Here we propose a model for SHP2 expression in which NF-κB and SHP2 progressively accumulate during the early stages of tumor development in liver tissues with a heterogeneous background, inflammation, and/or compensatory hepatocyte proliferation. After HCC initiation, SHP2 depletion could skew the balance between proliferation and inflammation and lead to an inflammatory phenotype involving STAT3, which might accelerate HCC development, as reported by Bard-Chapeau *et al* [26]. The HBx–NF-κB–SHP2 pathway might not be essential for the proliferation of hepatocytes that undergo malignant conversion; therefore, SHP2 expression might decline after HCC development. In this respect, we believe that SHP2 is a tumor suppressor, although its role is largely determined by NF-κB activity (Figure 7).

Recently, Jiang *et al*. reported differences in SHP2 expression between tumor (T) and adjacent non-tumor (NT) tissues and divided HCCs into two groups depending on the level of SHP2 expression: T < NT and T ≥ NT. In tumors, a strong decrease in SHP2 expression (T < NT) was associated with short overall survival [32]. We also found that SHP2 expression was lower in the 162 HCC tissues than in the matched non-neoplastic surrounding tissues (*P* < 0.001) (Figure 5B), but did not detect a relationship between SHP2 expression and the overall patient survival duration (*P* = 0.092). Instead, we found strong associations of SHP2 overexpression with a lack of microvascular invasion (*P* = 0.016) (Table 2) and the presence of advanced background tissue fibrosis (*P* = 0.015) (Figure 5C). SHP2 expression levels were very low in normal liver tissues from HBV-free individuals, but they gradually increased in HBV-infected livers in accordance with increased inflammation and fibrosis. In addition, SHP2 expression tended to decrease or disappear in tumors and increase in fibrotic background tissues. Taken together, our results indicate that SHP2, as a target of NFκB, is likely to be a marker of inflammation, the expression of which can be either maintained or increased during fibrosis and the early stages of HCC development. It is important to note that most studies of HCC development have involved specific conditions (such as LPS treatment, chemically induced HCC animal models, or knockout mice), which focus on the process of HCC development. Our *in vitro* data show the competition between HBx–NF-κB–SHP2 activity and STAT3 signaling; in contrast, our *in vivo* data are indicative of the consequences of competition between the two pathways.

In summary, we showed that SHP2 expression was activated by the HBx–NF-κB pathway. In patients with HCC, a loss of SHP2 expression was associated with suppressed NF-κB–SHP2–ERK pathway activity and accelerated HCC development, whereas SHP2 overexpression in parallel with increased STAT3 activity was associated with fibrosis promotion during the early stages of HCC development. To our knowledge, this is the first report to demonstrate the existence of an HBx–NF-κB–SHP2 pathway in HBV-infected livers and the simultaneous activation of SHP2 and STAT3 pathways in inflammatory livers, leading to the spontaneous development of HCC.

## MATERIALS AND METHODS

### Materials

EGF was obtained from Cell Signaling Technology (Beverly, MA, USA). An NF-κB inhibitor, parthenolide, was obtained from Sigma-Aldrich (P0667; St. Louis, MO, USA) and prepared in dimethyl sulfoxide.

### Cell culture

The HCC cell lines Huh7 and Hep3B were obtained from the National Cancer Institute (Bethesda, MD, USA). All cells were maintained in DMEM (Invitrogen-Gibco, Carlsbad, CA, USA) containing 10% fetal bovine serum, penicillin (100 U/ml), and streptomycin (100 μg/ml; Invitrogen-Gibco) at 37°C in a humidified 5% CO_2_ incubator. Huh7-X cells overexpressing HBx were maintained in DMEM containing 200 mg/ml of G418 (Duchefa Biochemie, Haarlem, Netherlands) [43].

### Western blot analysis

Whole-cell lysates were prepared in a cell lysis buffer (Cell Signaling Technology) containing a protease (Tech & Innovation^TM^, Bucheon, Korea) and phosphatase (sc-45065; Santa Cruz Biotechnology, Dallas, TX, USA) inhibitor cocktail. Proteins were separated on 10% or 12% SDS-PAGE gels, transferred to polyvinylidene fluoride membranes using an iBlot^TM^ dry blotting system (Invitrogen), and analyzed using primary antibodies against the following proteins: SHP2 (sc-280; Santa Cruz), ERK (sc-154; Santa Cruz), phospho-ERK (Tyr204) (sc-7383; Santa Cruz), HA (H6908; Sigma-Aldrich), Gab1 (06-579; Millipore, Billerica, MA, USA), GAP (06-157; Upstate Biotechnology, Charlottesville, VA, USA), EGFR (2232; Cell Signaling Technology), STAT3 (9132; Cell Signaling), phospho-STAT3 (Tyr705) (9131; Cell Signaling), and β-actin (A5441; Sigma-Aldrich) or vinculin (v9131; Sigma-Aldrich). Blots were developed using the SuperSignal West Pico chemiluminescent substrate (Thermo Scientific, Rockford, IL, USA).

### Quantitative real-time reverse transcription-polymerase chain reaction (qRT-PCR)

Total RNA (1 μg) was extracted using the NucleoSpin^®^ RNA II kit (740955; Macherey-Nagel, Duren, Germany) and reverse-transcribed using SuperScript^®^ II Reverse Transcriptase (Invitrogen). qRT-PCR was performed on a Bio-Rad iQ5 machine (Hercules, CA, USA) with SYBR Green (S7563; Invitrogen) and the following primer sets: HBx, 5′-GGACTCTACCGTCCCCTTCT-3′ and 5′-CGCTGAGAGTCCAAGAGTCC-3′, yielding a 203-bp fragment; SHP2, 5′-ATACGACGTTGGTGGAGGAG-3′ and 5′-TAAGGGGCTGCTTGAGTTGT-3′, yielding a 116-bp fragment; and GAPDH, 5′-GAAGGTGAAGGTCGG AGTC-3′ and 5′-GAAGATGGTGATGGGATTTC-3′, yielding a 226-bp product. The thermal cycling conditions comprised an initial denaturation step at 95°C for 5 min and 40 cycles at 95°C for 30 s, 60°C for 30 s, and 72°C for 30 s, followed by a final extension step at 72°C for 7 min. The expression of HBx or SHP2 mRNA was normalized to that of GAPDH.

### Luciferase assays

HEK293 cells were plated in 12-well culture plates and transfected with 0.5 μg of *Shp2* promoter-luciferase reporter plasmids (Shp2-Luc: 5′-flanking region of the human Shp2 promoter spanning nucleotides −1,727 to −151, with numbering starting at the position of the translation start site) or the control pGL3-Basic Vector (Promega, Madison, WI, USA) alone or in combination with 0.5 μg of a plasmid encoding HA–HBx or NF-κB, together with 0.01 μg of *Renilla* luciferase plasmid (pRL-SV40). Lipofectamine 2000 (Invitrogen) was used for transfection. The cells were harvested 48 h after transfection, and firefly luciferase activity was measured in three independent experiments. Data were normalized to *Renilla* luciferase activity.

### ChIP assay

Approximately 10^6^ Huh7 or HEK293 cells were plated onto 10-cm plates and transfected with 6 μg of HBx expression vectors. After a 24-h transfection step, the cells were cross-linked with 1% formaldehyde for 10 min at room temperature. ChIP assays were performed using an antibody against NF-κB–p65 (sc-372; Santa Cruz). Immunoprecipitated DNA was analyzed by PCR using primers spanning the NF-κB binding site: 5′-TGGCTTAAGCGAACCTCTTG-3′ and 5′-CGCTCCACTCATTAGCTGTG-3′. The GAPDH gene was used as an internal control and amplified using the following primers: 5′-TACTAGCGGTTTTACGGGCG-3′ and 5′-TCGAACAGGAGGAGCAGAGAGCGA-3′.

### Electrophoretic mobility shift assays (EMSA)

Nuclear extracts were prepared from Huh7 and Huh7-X cells using NE-PER Nuclear and Cytoplasmic Extraction Reagents (78833, Thermo Scientific, Rockford, IL) and EMSA was performed using a LightShift® Chemiluminescent EMSA Kit (20148, Thermo Scientific) according to the manufacturer's instructions. Eight micrograms of nuclear extracts were pre-incubated for 20 min at room temperature in a 20-μL reaction solution containing 1× binding buffer, 50 ng/μL poly (dI-dC), and 20 pmol of biotin-labeled probes complementary to the NF-κB–binding site (GGTAATTTCC) in the Shp2 promoter. Complexes were resolved in 6% non-denaturing polyacrylamide gels. For competition experiments, the nuclear extracts were pre-incubated for 10 min in the presence of a 1-, 10-, or 100-fold excess of the following unlabeled double-stranded oligonucleotides: wild-type NF-κB, 5′-GGCTAGGTAATTTCCCTGGTCAT-3′; mutated NF-κB, 5′-GGCTAGGaAcgTTCCCTGGTCAT-3′ (NF-κB–binding sites are underlined, and mutated sites are in lowercase).

### siRNA transfection

Hep3B cells were transfected with 25, 50 or 100 nmol/L siRNA targeting NF-κB (#6261, Cell Signaling) or with control siRNA (D-001210-01; GE Healthcare Dharmacon, Lafayette, CO) using Oligofectamine (Invitrogen). After 48 h, the cells were harvested.

### Immunoprecipitation experiment

Huh7-X and Hep3B cells were treated with EGF (50 ng/ml) for 10 min after serum starvation for 24 h. The cells were lysed in CHAPS lysis buffer containing 150 mM NaCl, 10 mM HEPES pH 7.5, 1% CHAPS, and protease inhibitors. Gab1 was precipitated from cell extracts during an overnight incubation with anti-Gab1 antibodies, after which 20 μl of protein A/G PLUS-agarose beads (sc-2003; Santa Cruz) were added. The immunoprecipitates were centrifuged and subjected to Western blot analysis with SHP2, EGFR, or Gab1 antibodies.

### ELISA

The levels of NF-κB-p65 and phospho-STAT3 (p-Tyr^705^) in tumors and background non-neoplastic liver tissues from patients with HCC were analyzed using the NBP2-29661 (Novus Biologicals, Littleton, CO, USA) and RAB0447 ELISA kits (Sigma, St. Louis, MO, USA), respectively. The tissues were lysed with a RIPA cell lysis buffer supplemented with phosphatase inhibitors. To detect NF-κB-p65 and phospho-STAT3 (p-Tyr^705^) proteins, ELISA plates were coated with a capture antibody and incubated overnight at 4°C. The wells were subsequently washed, blocked for 1 h, and incubated for 2 h with standards or samples. After extensive washing, the wells were incubated with a detection antibody followed by an HRP-conjugated secondary antibody for 1 h each. Color reactions were obtained by adding a substrate, followed by a stop solution. The absorbance of the color reactions was read at 450 nm using an absorbance spectrometer (Victor X3; PerkinElmer, Waltham, MA, USA).

### Isolation of proteins from HBV-infected HCC tissues

This work was approved by the Institutional Review Board of Asan Medical Center, and all patients provided written informed consent. A total of 23 pairs of HCC and peripheral non-neoplastic tissues from patients with HBV-associated HCC and their associated data were provided by Asan Bio-Resource Center [2011-2(31)], Korea Biobank Network at Asan Medical Center. All tissues were lysed with RIPA buffer [50 mM Tris-HCl (pH 8.0), 150 mM NaCl, 0.5 mM EDTA, 1 mM DTT, 0.1% NP-40, 0.1% SDS], followed by three freeze-thaw cycles and vortexing for 10 min at 4°C. After centrifugation at 16,100 ×*g* for 30 min, a volume of the upper layer, corresponding to 30 μg of the protein, was analyzed by Western blotting.

### Tissue microarrays

A total of 18 tissue microarrays were constructed using formalin-fixed, paraffin-embedded tissues from surgically resected or ultrasound-guided needle biopsy specimens. Hematoxylin and eosin-stained slides were examined by two pathologists (EY and HJK) to identify tumor and normal tissues and evaluate inflammation and fibrosis. Two tumor cores measuring 2 mm in diameter were arrayed from the corresponding paraffin blocks into a recipient block containing holes created using an arraying machine (TMArrayer; Pathology Devices, Westminster, MD, USA).

### IHC

IHC staining was performed on formalin-fixed, paraffin-embedded tissue sections using a Benchmark automatic immunostaining device (Ventana Medical Systems, Tucson, AZ, USA). Briefly, 5-μm-thick tissue sections were transferred onto adhesive slides and dried at 62°C for 30 min. After incubation in 3% H_2_O_2_ to block endogenous peroxidase activity, tissue sections were subjected to standard heat-mediated epitope retrieval in ethylene diamine tetraacetic acid (pH 8.0) for 32 min. The samples were incubated with primary antibodies against SHP2 (1:75; Abcam, Cambridge, UK) and phospho-STAT3 (Tyr705) (1:200, Cell signaling) for 16 min, respectively. SHP2– and phospho-STAT3–antibody complexes were detected with the OptiView DAB IHC Detection Kit (Ventana Medical Systems). Immunostained sections were counterstained with hematoxylin, dehydrated in ethanol, and cleared in xylene. The percentage of stained cells (0–100%) was used as an estimate of the SHP2 expression level. Tissue samples exhibiting diffuse cytoplasmic staining with/without nuclear staining in >10% of tumor cells or background non-neoplastic liver cells were considered SHP2 positive. Cases exhibiting strong nuclear staining in >10% of tumor cells or background non-neoplastic liver cells were considered positive for phospho-STAT3.

### Statistics

Values are presented as means ± standard deviations (*n* = 3 or 4). Comparisons between groups were analyzed using two-tailed Student's *t*-tests or the Wilcoxon rank sum test as appropriate. The relationship between SHP2 positivity and clinicopathologic factors was determined using the chi square test. Survival analysis was determined according to the Kaplan–Meier method, and survival curves were compared using the log-rank test. The Wilcoxon-matched pairs signed rank test was used to evaluate patients with matched tumor and normal tissues. Statistical significance (*P* < 0.05) is indicated in figures by asterisks (*). Statistical analyses were performed using the R program (v2.14.2, www.r-project.org, Vienna Austria) and the Stata/IC statistical software package (v12; StataCorp Ltd., College Station, TX, USA).

## SUPPLEMENTARY MATERIALS TABLES






