# Supplementary material for: SHP2 is induced by the HBx-NF-κB pathway and contributes to fibrosis during human early hepatocellular carcinoma development
Source: Oncotarget. 2017 Mar 6;8(16):27263–76. doi: 10.18632/oncotarget.15930 (PMC5432333; doi:10.18632/oncotarget.15930)
Supplement: Supplementary file 1 [file oncotarget-08-27263-s001.pdf]

SHP2 is induced by the HBx-NF-κB pathway and contributes to fibrosis during human early hepatocellular carcinoma development

SUPPLEMENTARY MATERIALS

SUPPLEMENTARY TABLES

Supplementary Table 1: List of clinicopathological features in 162 cases of HCCs

See Supplementary File 1

Supplementary Table 2: List of characteristics associated with SHP2 expression in 120 patients without HCCs

See Supplementary File 2
