# Supplementary material for: SHP2 is induced by the HBx-NF-κB pathway and contributes to fibrosis during human early hepatocellular carcinoma development
Source: Oncotarget. 2017 Mar 6;8(16):27263–76. doi: 10.18632/oncotarget.15930 (PMC5432333; doi:10.18632/oncotarget.15930)
Supplement: Supplementary file 2 [file oncotarget-08-27263-s002.docx]

Supplementary Table 1.

| ID | Age | Sex | AFP  (ng/ml) | HBV | Fibrosis stage | Microvascular invasion | Size  (cm) | Edmondson  grade | SHP2 expression in HCC  (%) | SHP2 expression in background liver (%) |
| --- | --- | --- | --- | --- | --- | --- | --- | --- | --- | --- |
| 1 | 41 | M | 55.7 | + | IV | No | 3.5 | II | 17.5 | 60 |
| 2 | 50 | M | 317 | + | IV | No | 2.2 | II | 0 | 80 |
| 3 | 57 | M | 3150 | + | IV | Yes | 2.5 | II | 2.5 | 80 |
| 4 | 59 | M | 341 | + | IV | No | 1.2 | III | 5 | - |
| 5 | 48 | M | 1.7 | + | III | No | 1.5 | II | 27.5 | 80 |
| 6 | 55 | M | 1.9 | + | III | No | 2 | I | 100 | 55 |
| 7 | 48 | M | 1150 | + | II | Yes | 1.5 | II | 0 | 0 |
| 8 | 56 | M | 251 | + | IV | No | 4 | III | 0 | 100 |
| 9 | 46 | M | 2.5 | + | III | Yes | 3 | III | 27.5 | 80 |
| 10 | 63 | M | 4.4 | + | IV | Yes | 4 | III | 0 | 95 |
| 11 | 65 | M | 1.4 | + | IV | No | 3.8 | II | 0 | 100 |
| 12 | 50 | M | 560 | + | III | No | 2.3 | III | 0 | 25 |
| 13 | 69 | F | 39700 | + | II | Yes | 7 | III | 7.5 | 60 |
| 14 | 56 | M | 270 | + | IV | Yes | 3 | III | 0 | 75 |
| 15 | 44 | M | 2710 | + | IV | No | 3 | II | 22.5 | 10 |
| 16 | 60 | F | 46.8 | + | II | No | 7 | II | 0 | 35 |
| 17 | 63 | M | 1.9 | + | IV | Yes | 8 | III | 16.5 | 10 |
| 18 | 48 | M | 2 | + | IV | No | 3.5 | I | 0 | 0 |
| 19 | 61 | M | 5.7 | + | IV | No | 2 | II | 0 | 100 |
| 20 | 50 | F | 4200 | + | IV | Yes | 5 | II | 0 | 90 |
| 21 | 67 | M | 3.7 | + | III | Yes | 3.3 | II | 17.5 | 65 |
| 22 | 62 | M | 195 | + | IV | Yes | 5.8 | II | 0 | 15 |
| 23 | 58 | M | 16.7 | + | III | No | 4 | II | 0 | 40 |
| 24 | 53 | F | 182 | + | IV | No | 2.7 | II | 0 | 65 |
| 25 | 62 | M | 9.9 | + | IV | No | 3.5 | II | 0 | 0 |
| 26 | 58 | M | 7.6 | + | IV | No | 2 | II | 0 | 80 |
| 27 | 52 | M | 8.1 | + | IV | No | 2.7 | III | 0 | 60 |
| 28 | 47 | M | 1.5 | + | III | Yes | 3.8 | II | 0 | 65 |
| 29 | 66 | M | 13.5 | + | III | No | 2.5 | II | 0 | 65 |
| 30 | 46 | M | 5 | + | IV | No | 2.5 | II | 2.5 | 70 |
| 31 | 46 | M | 82.7 | + | IV | Yes | 3.5 | III | 0 | 0 |
| 32 | 51 | M | 9.8 | + | IV | Yes | 5.8 | IV | 0 | 65 |
| 33 | 69 | M | 72.8 | + | IV | No | 2.5 | II | 0 | 0 |
| 34 | 53 | M | 21.9 | + | IV | Yes | 2.2 | II | 2.5 | 80 |
| 35 | 37 | M | 7890 | + | III | Yes | 5 | II | 92.5 | 75 |
| 36 | 59 | M | 72 | + | II | No | 4.5 | II | 0 | 0 |
| 37 | 34 | F | 2780 | + | I | Yes | 5 | II | 0 | 1 |
| 38 | 57 | F | 353 | + | IV | Yes | 2.1 | III | 0 | 0 |
| 39 | 67 | M | 5.2 | + | III | No | 4.2 | II | 5 | 25 |
| 40 | 43 | M | 157 | + | IV | No | 4.7 | II | 2.5 | 55 |
| 41 | 44 | M | 204 | + | III | No | 3.5 | III | 5 | 65 |
| 42 | 41 | M | 108 | + | II | Yes | 8.5 | II | 0 | 0 |
| 43 | 53 | M | 22.2 | + | IV | Yes | 2.5 | II | 0 | 90 |
| 44 | 53 | M | 13 | + | IV | No | 3 | III | 30 | 60 |
| 45 | 71 | F | 1.5 | + | I | No | 9 | III | 0 | 90 |
| 46 | 56 | M | 23.6 | + | I | No | 4.5 | II | 90 | 10 |
| 47 | 51 | M | 5 | + | IV | No | 4.5 | II | 0 | - |
| 48 | 57 | F | 323 | + | IV | No | 8 | III | 0 | - |
| 49 | 37 | M | 472000 | + | III | Yes | 12.5 | III | 0 | - |
| 50 | 54 | M | 5.2 | + | IV | No | 4 | III | 0 | 85 |
| 51 | 55 | M | 3.6 | + | IV | No | 2.5 | III | 0 | 80 |
| 52 | 66 | F | 4.5 | + | IV | No | 3.5 | II | 0 | 40 |
| 53 | 55 | M | 8040 | + | III | Yes | 5.5 | III | 0 | 90 |
| 54 | 64 | M | 43300 | + | IV | No | 6 | II | 2.5 | 90 |
| 55 | 53 | M | 225 | + | III | No | 2.5 | II | 5 | 55 |
| 56 | 46 | M | 2.9 | + | I | No | 6.5 | II | 92.5 | 30 |
| 57 | 49 | M | 5.3 | + | IV | Yes | 3.5 | III | 0 | 35 |
| 58 | 53 | M | 12.1 | + | I | No | 14 | II | 42.5 | - |
| 59 | 50 | F | 1.8 | + | I | No | 4 | II | 45 | 30 |
| 60 | 57 | M | 11.6 | + | IV | No | 2 | II | 0 | 100 |
| 61 | 34 | M | 2.1 | + | IV | No | 1.8 | II | 27.5 | 90 |
| 62 | 41 | M | 1350 | + | II | No | 9.3 | II | 15 | 80 |
| 63 | 42 | F | 4900 | + | III | No | 4.5 | II | 12.5 | 35 |
| 64 | 49 | M | 596 | + | II | Yes | 9 | III | 0 | 55 |
| 65 | 40 | M | 4.3 | + | II | No | 4 | II | 10 | 90 |
| 66 | 26 | M | 156 | + | IV | No | 4 | II | 0 | 70 |
| 67 | 51 | M | 9.5 | + | III | No | 3.2 | II | 0 | 85 |
| 68 | 48 | F | 123 | + | III | Yes | 8 | III | 0 | 0 |
| 69 | 40 | M | 5.3 | + | IV | Yes | 4.2 | II | 0 | 30 |
| 70 | 45 | M | 226 | + | III | No | 4 | II | 0 | - |
| 71 | 57 | M | 372 | + | IV | No | 3 | III | 0 | 15 |
| 72 | 55 | M | 17.4 | + | IV | No | 3.5 | II | 12.5 | 70 |
| 73 | 35 | F | 38900 | + | III | Yes | 16 | III | 0 | 35 |
| 74 | 54 | F | 102 | + | IV | No | 2.5 | II | 42.5 | 60 |
| 75 | 45 | M | 38.1 | + | IV | No | 4.5 | III | 0 | 10 |
| 76 | 60 | F | 1.3 | + | III | No | 3.2 | II | 22.5 | 0 |
| 77 | 30 | F | 3940 | + | II | Yes | 7 | III | 0 | 5 |
| 78 | 64 | M | 31100 | + | IV | No | 3.8 | II | 92.5 | 40 |
| 79 | 46 | M | 4270 | + | IV | No | 3.1 | II | 0 | 5 |
| 80 | 52 | M | 2.1 | + | III | Yes | 4.2 | II | 0 | 95 |
| 81 | 59 | F | 79900 | + | IV | Yes | 8 | II | 25 | 85 |
| 82 | 42 | M | 2.4 | + | I | No | 4 | II | 20 | 10 |
| 83 | 47 | M | 73.8 | + | IV | No | 4.5 | IV | 7.5 | 35 |
| 84 | 41 | F | 314 | + | III | Yes | 6 | III | 0 | 40 |
| 85 | 46 | M | 230 | + | III | Yes | 7.5 | III | 0 | 75 |
| 86 | 51 | M | 1.2 | + | III | No | 6.5 | II | 0 | 85 |
| 87 | 61 | M | 9.3 | + | IV | No | 7.6 | II | 12.5 | 90 |
| 88 | 49 | F | 519 | + | IV | No | 3.6 | I | 62.5 | 90 |
| 89 | 57 | M | 3.8 | + | III | No | 3.3 | II | 2.5 | 15 |
| 90 | 43 | M | 4.4 | + | II | No | 12 | II | 30 | 55 |
| 91 | 37 | M | 1665.4 | + | III | Yes | 3.5 | III | 0 | 0 |
| 92 | 56 | M | 9.4 | + | IV | No | 5 | II | 0.5 | 90 |
| 93 | 53 | M | 42.4 | + | III | No | 5 | II | 85 | 90 |
| 94 | 42 | F | 4960 | + | III | Yes | 4 | III | 0 | 95 |
| 95 | 54 | F | 1640 | + | IV | Yes | 3 | II | 0 | 70 |
| 96 | 54 | F | 7500 | + | IV | No | 3.5 | II | 0 | 75 |
| 97 | 44 | M | 350 | + | III | No | 6.5 | III | 10 | 80 |
| 98 | 62 | F | 894 | + | IV | No | 3.5 | II | 5 | 0 |
| 99 | 57 | M | 4310 | + | IV | No | 3.5 | II | 35 | 5 |
| 100 | 63 | M | 2600 | + | II | No | 4 | II | 0 | 95 |
| 101 | 55 | M | 49.9 | + | IV | No | 5 | II | 0 | 75 |
| 102 | 59 | M | 3.6 | + | II | No | 3 | II | 0 | 5 |
| 103 | 66 | M | 43.1 | + | III | No | 3 | II | 40 | 5 |
| 104 | 56 | F | 83.6 | + | III | No | 4.3 | II | 0 | 60 |
| 105 | 49 | M | 1.7 | + | IV | No | 2 | II | 0 | 70 |
| 106 | 43 | F | 2380.3 | + | III | No | 2.9 | II | 1.5 | 85 |
| 107 | 61 | M | 6.2 | + | IV | No | 5.3 | II | 5 | 80 |
| 108 | 66 | M | 4.4 | + | III | No | 5 | II | 0 | 20 |
| 109 | 57 | F | 1.7 | + | IV | No | 1.8 | II | 0 | 0 |
| 110 | 54 | M | 192 | + | IV | No | 1.7 | III | 0 | 20 |
| 111 | 44 | M | 771 | + | II | No | 2.6 | II | 0 | 1 |
| 112 | 61 | M | 76 | + | III | No | 2.3 | II | 0 | 75 |
| 113 | 68 | F | 894.1 | + | III | Yes | 2 | III | 0 | 75 |
| 114 | 51 | M | 303 | + | IV | Yes | 3.3 | II | 87.5 | 100 |
| 115 | 52 | F | 142 | + | IV | No | 3.7 | III | 0.5 | 35 |
| 116 | 60 | M | 4.9 | + | I | No | 7.2 | II | 0 | 45 |
| 117 | 61 | M | 126 | + | IV | No | 3 | II | 12.5 | 55 |
| 118 | 45 | M | 1442.4 | + | IV | Yes | 2.8 | II | 0 | 1 |
| 119 | 61 | F | 368.6 | + | IV | No | 2.8 | III | 0 | 1 |
| 120 | 41 | M | 457.6 | + | III | Yes | 3.8 | II | 0 | 75 |
| 121 | 53 | F | 1190.7 | + | IV | No | 2.5 | III | 0 | 85 |
| 122 | 47 | M | 3.3 | + | IV | No | 1.3 | IV | 35 | 55 |
| 123 | 53 | M | 3560 | + | IV | No | 3.3 | II | 22.5 | 75 |
| 124 | 59 | M | 975.6 | + | IV | Yes | 6 | IV | 0 | 85 |
| 125 | 40 | F | 615 | + | III | No | 5.5 | II | 0 | 80 |
| 126 | 47 | M | 6.1 | + | I | No | 2.8 | II | 0 | 40 |
| 127 | 53 | M | 779140 | + | IV | Yes | 12.8 | II | 0 | 40 |
| 128 | 51 | M | 18189.8 | + | IV | Yes | 8.9 | III | 0 | 85 |
| 129 | 60 | M | 122.1 | + | IV | No | 2.2 | III | 0 | 80 |
| 130 | 62 | F | 86.4 | + | IV | No | 4 | III | 10 | 10 |
| 131 | 69 | M | 11.5 | + | IV | No | 2.2 | II | 0 | 95 |
| 132 | 43 | M | 174 | + | IV | No | 1.5 | II | 0 | 0 |
| 133 | 51 | M | 347.5 | + | IV | Yes | 3.5 | III | 0 | 90 |
| 134 | 54 | F | 530 | + | IV | No | 3 | II | 0 | 85 |
| 135 | 44 | F | 92.7 | + | IV | No | 3.5 | II | 0 | 95 |
| 136 | 40 | M | 12.1 | + | IV | No | 4 | II | 2.5 | 60 |
| 137 | 68 | M | 660 | + | IV | No | 4 | II | 0 | 75 |
| 138 | 58 | F | 68.7 | + | IV | No | 1.5 | III | 0 | 0 |
| 139 | 62 | M | 10.1 | + | II | No | 3.4 | III | 67.5 | 0 |
| 140 | 48 | F | 19.8 | + | III | No | 2.8 | II | 0 | 75 |
| 141 | 57 | F | 1094.7 | + | IV | No | 4 | II | 0 | 65 |
| 142 | 43 | M | 4.8 | + | III | Yes | 2 | III | 2.5 | 5 |
| 143 | 51 | M | 3.8 | + | IV | No | 2.8 | II | 15 | 85 |
| 144 | 68 | F | 3.9 | + | II | No | 1.5 | II | 0 | 25 |
| 145 | 55 | M | 2.6 | + | III | No | 3 | II | 62.5 | 70 |
| 146 | 52 | M | 7680 | + | IV | Yes | 6 | IV | 0 | 60 |
| 147 | 67 | M | 1.4 | + | III | Yes | 4.8 | II | 0 | 90 |
| 148 | 46 | F | 8.9 | + | IV | No | 5 | II | 0 | 0 |
| 149 | 54 | M | 5.4 | + | II | No | 2.2 | III | 0 | 85 |
| 150 | 51 | F | 8.9 | + | III | Yes | 5.5 | III | 0 | 25 |
| 151 | 51 | M | 1359.7 | + | IV | Yes | 1.9 | III | 0 | 25 |
| 152 | 53 | M | 11.7 | + | IV | No | 2.8 | II | 20 | 60 |
| 153 | 64 | M | 1133.7 | + | IV | No | 1.5 | III | 32.5 | 0 |
| 154 | 57 | F | 6.7 | + | IV | No | 3.8 | II | 0 | 5 |
| 155 | 42 | M | 2830 | + | IV | No | 4.7 | II | 0 | 0 |
| 156 | 48 | M | 3.9 | + | III | No | 2.2 | II | 0 | 80 |
| 157 | 61 | M | 2 | + | II | No | 3.3 | II | 20 | 0 |
| 158 | 64 | M | 1.1 | + | III | No | 2.5 | III | 0 | 90 |
| 159 | 64 | M | 9.5 | + | III | Yes | 2.2 | II | 0 | 0 |
| 160 | 51 | F | 214 | + | IV | No | 1.5 | II | 0 | 0 |
| 161 | 52 | M | 1040 | + | IV | Yes | 12.4 | II | 0 | 100 |
| 162 | 62 | M | 238 | + | IV | Yes | 9 | III | 0 | 0 |
