# Supplementary material for: SHP2 is induced by the HBx-NF-κB pathway and contributes to fibrosis during human early hepatocellular carcinoma development
Source: Oncotarget. 2017 Mar 6;8(16):27263–76. doi: 10.18632/oncotarget.15930 (PMC5432333; doi:10.18632/oncotarget.15930)
Supplement: Supplementary file 3 [file oncotarget-08-27263-s003.docx]

Supplementary Table 2.

| **Dysplastic nodule** | | | | | | | |
| --- | --- | --- | --- | --- | --- | --- | --- |
| ID | Age | Sex | AFP | HBV | SHP2 expression in dysplastic nodule 1 (%) | SHP2 expression in dysplastic nodule 2 (%) | SHP2 expression in cirrhotic background liver (%) |
| 1 | 64 | F | 7.2 | positive | 30 | 30 | 20 |
| 2 | 60 | F | 2.2 | positive | 40 | 40 | 40 |
| 3 | 54 | M | 14.4 | positive | 95 | 95 | 100 |
| 4 | 53 | M | 24.2 | positive | 100 | 100 | 100 |
| 5 | 53 | M | 2.4 | positive | 100 | 100 | 100 |
| 6 | 53 | M | 1310 | positive | 20 | 15 | 0 |
| 7 | 50 | M | 4.3 | positive | 80 | 90 | 100 |
| 8 | 61 | F | 577 | positive | 0 | 0 | 100 |
| 9 | 50 | M | 35.3 | positive | 100 | 100 | 70 |
| 10 | 50 | M | 12.7 | positive | 100 | 100 | 90 |
| 11 | 53 | M | 1.5 | positive | 50 | 30 | 95 |
| 12 | 47 | M | 7.4 | positive | 0 | 0 | 80 |
| 13 | 60 | M | 10.5 | positive | 100 | 100 | 100 |
| 14 | 41 | M | 23.3 | positive | 85 | 85 | 70 |
| 15 | 50 | M | 454.8 | positive | 90 | 95 | 90 |
| 16 | 60 | M | 17.4 | positive | 100 | 100 | 50 |
| 17 | 55 | M | 7.9 | positive | 0 | 0 | 5 |
| 18 | 50 | M | 12 | positive | 60 | 20 | 10 |
| 19 | 54 | M | 477 | positive | 90 | 80 | 40 |
| 20 | 48 | M | 9.8 | positive | 50 | 15 | 20 |
| 21 | 60 | M | 10.4 | positive | 0 | 10 | 10 |
| 22 | 67 | M | 5.6 | positive | 40 | 60 | 50 |
| 23 | 53 | M | 34.3 | positive | 70 | 80 | 85 |
| 24 | 58 | F | 55.3 | positive | 70 | 80 | 80 |
| 25 | 57 | M | 5.1 | positive | 15 | 30 | 60 |
| 26 | 61 | M | 68.5 | positive | 5 | 0 | 100 |
| 27 | 46 | M | 7 | positive | 70 | 70 | 20 |
| 28 | 54 | M | 2.7 | positive | 15 | 0 | 0 |
| 29 | 63 | M | 6.2 | positive | 15 | 40 | 90 |
| 30 | 52 | F | 8.1 | positive | 100 | 30 | 15 |
| 31 | 50 | M | 2.6 | positive | 60 | 0 | 85 |
| 32 | 51 | M | 15.2 | positive | 5 | 5 | 20 |
| 33 | 45 | M | 32.2 | positive | 0 | 0 | 0 |
| 34 | 57 | M | 4.2 | positive | 30 | 5 | 0 |
| 35 | 59 | F | 909 | positive | 80 | 100 | 70 |
| 36 | 49 | F | 13.6 | positive | 10 | 10 | 40 |
| 37 | 56 | M | 2.8 | positive | 100 | 100 | 80 |
| 38 | 52 | M | 114 | positive | 100 | 100 | 100 |
| 39 | 51 | M | 17.3 | positive | 40 | 30 | 5 |
| 40 | 60 | M | 5.1 | positive | 0 | 0 | 20 |
| 41 | 50 | M | 284 | positive | 30 | 5 | 50 |
| 42 | 50 | M | 39 | positive | 80 | 30 | 60 |
| 43 | 55 | M | 3.2 | positive | 90 | 90 | 70 |
| 44 | 71 | F | 24.7 | positive | 25 | 30 | 90 |
| 45 | 61 | F | 0.66 | positive | 80 | 90 | 95 |
| 46 | 67 | M | 5.2 | positive | 60 | 65 | 70 |
| 47 | 59 | M | 2 | positive | 30 | 50 | 30 |
| 48 | 60 | F | 32.9 | positive | 100 | 100 | 90 |
| 49 | 54 | F | 33.3 | positive | 40 | 20 | 10 |
| 50 | 58 | M | 4.4 | positive | 100 | 100 | 80 |
| 51 | 49 | M | 700 | positive | 100 | 50 | 60 |
| Chronic hepatitis | | | | | | | |
| ID | Age | Sex | AFP | HBV | SHP2 expression in chronic hepatitis liver 1 (%) | SHP2 expression in chronic hepatitis liver 2 (%) | - |
| 1 | 63 | M | - | positive | - | - | - |
| 2 | 30 | M | - | positive | 30 | - | - |
| 3 | 46 | M | - | positive | 40 | 15 | - |
| 4 | 49 | F | - | positive | - | - | - |
| 5 | 51 | F | - | positive | 10 | 0 | - |
| 6 | 44 | M | - | positive | 70 | 80 | - |
| 7 | 46 | M | - | positive | 30 | 40 | - |
| 8 | 19 | M | - | positive | 50 | 50 | - |
| 9 | 43 | M | - | positive | - | - | - |
| 10 | 67 | M | - | positive | 60 | 60 | - |
| 11 | 43 | M | - | positive | 5 | 5 | - |
| 12 | 33 | M | - | positive | 15 | - | - |
| 13 | 60 | F | - | positive | - | - | - |
| 14 | 28 | M | - | positive | 10 | 5 | - |
| 15 | 54 | M | - | positive | 20 | - | - |
| 16 | 31 | M | - | positive | 0 | 0 | - |
| 17 | 57 | F | - | positive | 0 | 0 | - |
| 18 | 59 | M | - | positive | 15 | 0 | - |
| 19 | 61 | F | - | positive | 0 | 0 | - |
| 20 | 51 | F | - | positive | 0 | 0 | - |
| 21 | 58 | M | - | positive | 10 | 15 | - |
| Normal | | | | | | | |
| ID | Age | Sex | AFP | HBV | SHP2 expression in normal liver 1 (%) | SHP2 expression in normal liver 2 (%) | - |
| 1 | 22 | M | - | negative | 0 | 0 | - |
| 2 | 26 | F | - | negative | 20 | 20 | - |
| 3 | 33 | F | - | negative | 0 | 0 | - |
| 4 | 22 | M | - | negative | - | 0 | - |
| 5 | 21 | M | - | negative | 0 | 0 | - |
| 6 | 24 | M | - | negative | 0 | 0 | - |
| 7 | 24 | M | - | negative | 50 | - | - |
| 8 | 34 | M | - | negative | 5 | 0 | - |
| 9 | 24 | M | - | negative | 10 |  | - |
| 10 | 15 | M | - | negative | 95 | 90 | - |
| 11 | 15 | M | - | negative | 0 | 0 | - |
| 12 | 22 | M | - | negative | - | - | - |
| 13 | 25 | M | - | negative | 40 | 50 | - |
| 14 | 34 | M | - | negative | 0 | 0 | - |
| 15 | 36 | M | - | negative | 20 | 20 | - |
| 16 | 39 | M | - | negative | 10 | 10 | - |
| 17 | 33 | M | - | negative | 0 | 0 | - |
| 18 | 22 | F | - | negative | 0 | 0 | - |
| 19 | 45 | F | - | negative | 0 | 0 | - |
| 20 | 38 | M | - | negative | 25 | 25 | - |
| 21 | 32 | F | - | negative | 50 | 50 | - |
| 22 | 17 | F | - | negative | 0 | 0 | - |
| 23 | 44 | M | - | negative | 80 | - | - |
| 24 | 18 | M | - | negative | 0 | - | - |
| 25 | 27 | M | - | negative | - | - | - |
| 26 | 22 | M | - | negative | 10 | 20 | - |
| 27 | 28 | M | - | negative | 0 | 0 | - |
| 28 | 33 | M | - | negative | 50 | 40 | - |
| 29 | 17 | F | - | negative | 40 | 35 | - |
| 30 | 33 | F | - | negative | 5 | 95 | - |
| 31 | 38 | M | - | negative | 0 | 0 | - |
| 32 | 37 | M | - | negative | 0 | 0 | - |
| 33 | 32 | M | - | negative | 20 | 15 | - |
| 34 | 26 | M | - | negative | 50 | 50 | - |
| 35 | 49 | M | - | negative | 0 | - | - |
| 36 | 44 | M | - | negative | 10 | - | - |
| 37 | 20 | M | - | negative | 15 | 20 | - |
| 38 | 23 | M | - | negative | 0 | 0 | - |
| 39 | 21 | M | - | negative | 5 | - | - |
| 40 | 21 | M | - | negative | 0 | 0 | - |
| 41 | 21 | M | - | negative | 0 | 0 | - |
| 42 | 45 | F | - | negative | 10 | - | - |
| 43 | 20 | M | - | negative | 0 | 0 | - |
| 44 | 28 | M | - | negative | 0 | 0 | - |
| 45 | 54 | M | - | negative | 0 | 0 | - |
| 46 | 27 | M | - | negative | 0 | 0 | - |
| 47 | 18 | M | - | negative | 10 | 5 | - |
| 48 | 28 | M | - | negative | - | 0 | - |
